# LIG4 syndrome: clinical and molecular characterization in a Chinese cohort

**DOI:** 10.1186/s13023-020-01411-x

**Published:** 2020-05-29

**Authors:** Bijun Sun, Qiuyu Chen, Ying Wang, Danru Liu, Jia Hou, Wenjie Wang, Wenjing Ying, Xiaoying Hui, Qinhua Zhou, Jinqiao Sun, Xiaochuan Wang

**Affiliations:** Department of Clinical Immunology, Children’s Hospital of Fudan University, 399 Wanyuan Road, Shanghai, 201102 China

**Keywords:** DNA ligase IV syndrome, Microcephaly, Inflammatory bowel disease, Immunodeficiency, Genetic testing

## Abstract

**Background:**

DNA Ligase IV (LIG4) syndrome is a rare disease with few reports to date. Patients suffer from a broad spectrum of clinical features, including microcephaly, growth retardation, developmental delay, dysmorphic facial features, combined immunodeficiency, and malignancy predisposition. There may be a potential association between genotypes and phenotypes. We investigated the characteristics of LIG4 syndrome in a Chinese cohort.

**Results:**

All seven patients had growth restriction. Most patients (6/7) had significant microcephaly (< − 3 SD). Recurrent bacterial infections of the lungs and intestines were the most common symptoms. One patient had myelodysplastic syndromes. One patient presented with an inflammatory bowel disease (IBD)-like phenotype. Patients presented with combined immunodeficiency. The proportions of naïve CD4+ and naïve CD8+ T cells decreased notably in five patients. All patients harbored compound heterozygous mutations in the *LIG4* gene, which consisted of a missense mutation (c.833G > T, p.R278L) and a deletion shift mutation, primarily c.1271_1275delAAAGA (p.K424Rfs*20). Two other deletion mutations, c.1144_1145delCT and c.1277_1278delAA, were novel. Patients with p.K424Rfs*20/p.R278 may have milder dysmorphism but more significant IgA/IgM deficiency compared to the frequently reported genotype p.R814X/p.K424Rfs*20. One patient underwent umbilical cord blood stem cell transplantation (UCBSCT) but died.

**Conclusions:**

The present study reported the clinical and molecular characteristics of a Chinese cohort with LIG4 syndrome, and the results further expand the phenotypic and genotypic spectrum and our understanding of genotype-to-phenotype correlations in LIG4 syndrome.

## Background

DNA ligase IV (LIG4) syndrome is an exceptionally rare autosomal recessive disorder that belongs to the group of hereditary diseases associated with impaired DNA damage-response mechanisms. The distinct features of LIG4 syndrome patients are microcephaly, growth retardation, developmental delay, dysmorphic facial features, variable immunodeficiency, pancytopenia, malignancy predisposition and pronounced clinical and cellular radiosensitivity [1].

DNA ligase IV functions in the nonhomologous end-joining (NHEJ) pathway, which is a major mechanism involved in the repair of DNA double-strand breaks (DSBs) in mammals [2], and it leads to an increased sensitivity to ionizing radiation. DSBs are also involved in the processes of class switch and V(D)J recombination during immune development. Therefore, failure to perform the processes caused by NHEJ deficiency confers (severe) combined immunodeficiency.

The *LIG4* gene maps to chromosome 13q33-q34, and it has a complex structure formed by four domains: the DNA-binding domain (DBD), adenylation domain (AdD), oligo-binding domain (OBD) and XRCC4-binding domain (XBD). LIG4 syndrome is caused by homozygous or compound heterozygous mutations in the *LIG4* gene, and the most common genotype is p.R814X/p.K424Rfs*20 [3]. Murray et al. [4] first proposed a genotype–phenotype correlation between the position of truncating mutations associated with p.R814X and disease severity.

Although this disease was first described nearly 30 years ago, only a few cases have been reported to date [3–27]. The present study reported the clinical, immunological and genetic characteristics of 7 Chinese patients with LIG4 syndrome.

## Methods

The Ethics Committee of the Children’s Hospital of Fudan University approved this study. Informed consent was obtained from the parents of the patients.

### Patients and clinical data

LIG4 syndrome was suspected based on the clinical manifestations of patients referred to our hospital between July 2014 and December 2019 and was further confirmed via immune function and gene detection in the present study. The relevant data are summarized in detail. Previous patients and mutations reported in PubMed Medline (https://www.ncbi.nlm.nih.gov/pubmed/) were reviewed and compared.

### Immunological function

Routine blood counts and immunological function analyses were performed. We used nephelometry to detect immunoglobulins, including IgG, IgA, and IgM, and lymphocyte subsets were measured using flow cytometry (Becton Dickinson, Franklin Lakes, NJ, USA). The following validated antibodies were used for flow cytometry: anti-CD3 (UCHT1), anti-CD8 (RPAT8), anti-CD27 (M-T271), anti-CD45RA (HI100), anti-CD4 (RPA-T4), anti-TCRαβ (T10B9.1A-31), anti-TCRγδ (B1), anti-CD19 (HIB19), anti-CD24 (ML5), anti-CD38 (HIT2), and anti-IgD (IA6–2) (all from BD Biosciences).

### Molecular analysis

Genomic DNA was extracted from the peripheral blood of the patients and their parents using the QIAamp DNA Blood Mini kit (Qiagen, Hilden, Germany). DNA quality was assessed using a NanoDrop ultraviolet spectrophotometer (Thermo Fisher Scientific, USA).

Next-generation sequencing was performed using a panel that included all previously reported immunodeficiency genes. Genomic DNA fragments of patients were ligated with adaptors so that two paired-end DNA libraries with insert sizes of 500 bp were formed for all samples. The DNA libraries after enrichment were sequenced on the HiSeq 2000 platform in accordance with the manufacturer’s instructions (Illumina, San Diego, CA). The variants were annotated in ANNOVAR and VEP software and predicted with SIFT, PolyPhen-2 and MutationTaster. The mutations were confirmed using Sanger sequencing.

## Results

### Clinical manifestations

#### Overview

Seven patients (4 males and 3 females) were diagnosed over a 5-year period in our center. The average age of morbidity was 5.3 months (range, 1 week-14 months), and the mean time of diagnosis was extended to 18.4 months. All of these cases were full-term infants. No patients were born out of consanguineous marriages. No disease-related family histories were found, except the mother of patient 2 (P2) and P5 had a previous pregnancy with embryo growth arrest. The clinical findings are summarized in Table 1.

**Table 1:** Baseline characteristics of patients with *LIG4* mutations

Patients	P1	P2	P3	P4	P5	P6	P7
Age at presentation	11 m	1 m	1w	4 m	1 m	14 m	6 m
Age of diagnosis	21 m	18 m	15 m	6 m	3 m	28 m	38 m
Sex	M	M	F	M	M	F	F
Birth Weight	2050 g	2770 g	2100 g	NA	2700 g	2550 g	2300 g
Family history	–	+	–	–	+	–	–
Microcephaly	+	+	+	+	+	–	+
Facial dysmorphism	–	–	–	–	–	–	–
Developmental retardation	+	–	+	–	–	–	–
Growth restriction	+	+	+	+	+	+	+
Clinical presentation	Diarrhea, thrombocytopenia, pneumonia, otitis media, thrush, vitiligo	Pneumonia, canker sores, recurrent fever, diarrhea, intestinal ulceration, impaired liver function	Omphalitis, pneumonia, thrush, leukopenia, canker sores, diarrhea, skin and soft tissue infection	Erythroderma, pneumonia, diarrhea, thrombocytopenia	Eczema, generalized lymphadenopathy	Pneumonia, thrush, canker sores, herpes simplex, diarrhea, pancytopenia	Recurrent upper respiratory tract infection, diarrhea, otitis media, pneumonia, thrombocytopenia, leukopenia
Pathogenic microorganism	Sputum: *Acinetobacter baumannii, Parainfluenza virus 3, Acinetobacter pittii, Candida albicans, Staphylococcus haemolyticus, Enterobacter cloacae, Klebsiella pneumoniae, Flavobacterium menigosepticum*	Sputum: *Respiratory syncytial virus*	Blood: *Staphylococcus hominis*; Excrement: *Salmonella typhimurium*	BALF: *Haemophilus influenzae, Pneumocystis, Flavobacterium menigosepticum*; Sputamentum: *Parainfluenza virus 3*	Negative	Excrement: *Salmonella enteritidis*	Sputum: *Pseudomonas aeruginosa*
Treatment	UCBSCT	Antibiotics, steroid	Antibiotics	Antibiotics	Antibiotics	Antibiotics	Antibiotics
Follow-up	Died	Died	Lost	Died	Died	Lost	Alive

The primary symptom was listed at first in the table of clinical presention

*M* Male, *F* Female, *NA* no available, *BALF* brocho-alveolar larage fluid, *UCBSCT* Umbilical cord blood stem cell transplantation

**Fig. 1:**
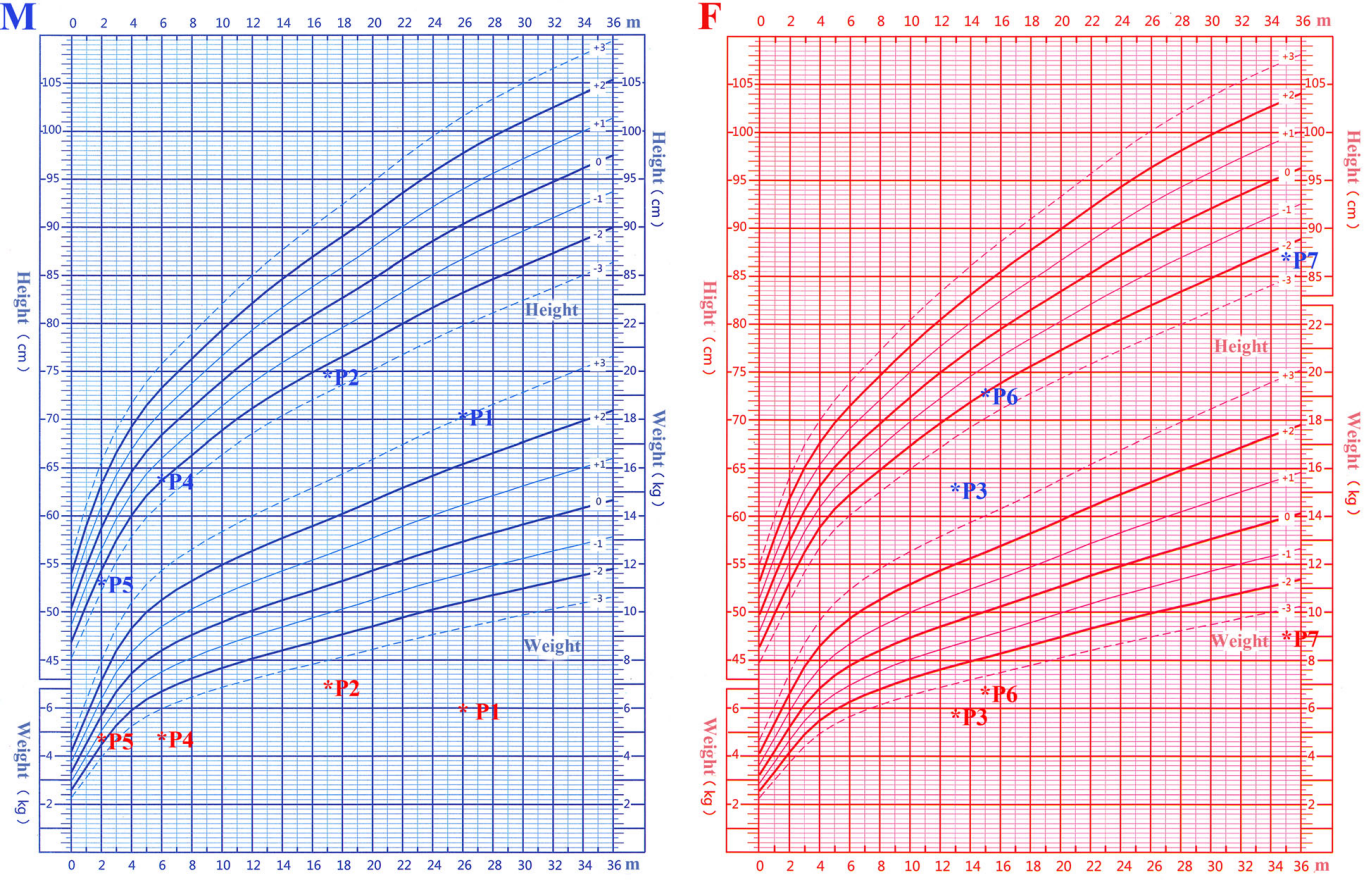
Growth index of patients with *LIG4* syndrome compared to healthy children in China. Height is marked in blue, and weight is marked in red. M: male. F: female

Microcephaly and growth restriction was obvious in these patients. The head circumference of six patients was more than 3 SD below the population mean of the same age and gender. One patient had a head circumference between 2 and 3 SD below the mean. Most of the patients had postnatal underweight (6 of 7 < − 3 SD) and stature lower than standard (2 of 7 < − 3 SD), and the weight loss was more pronounced (Fig. 1). Three full-term, small-for-gestational-age infants indicated intrauterine growth retardation during the early life stage. Two patients had clinical developmental retardation, which was primarily manifested as delayed language and motor retardation compared to healthy children of the same age. Head MRI of five patients showed no abnormalities. Intelligence assessment of scales or questionnaires was only completed in P1 and P2. However, the typical facial deformity was not found in our study.

#### Infection characteristics

**Fig. 2:**
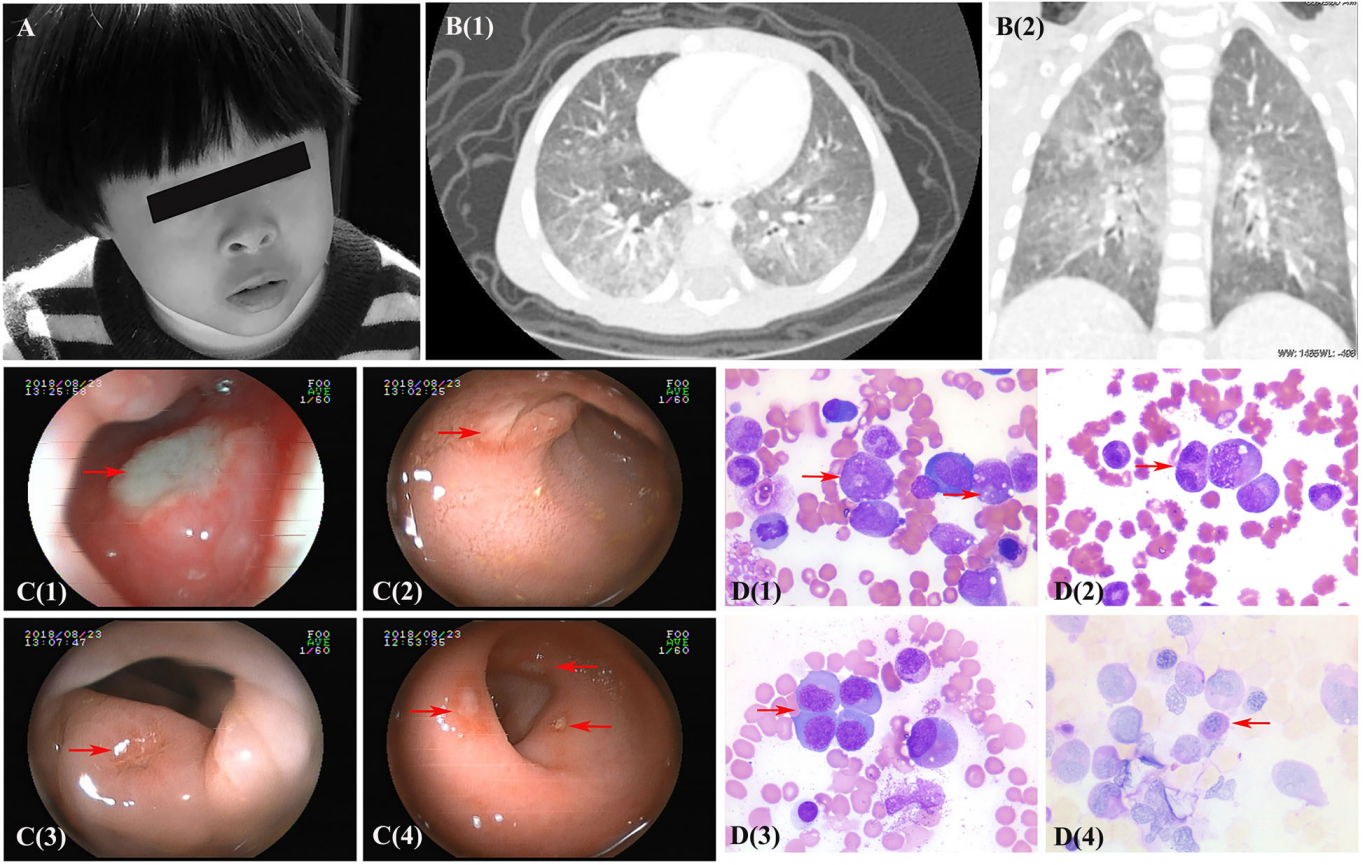
Clinical data of patients with LIG4 syndrome. **a**: Facial features of P7. **b**: The axial (1) and coronal (2) CT scan of the chest (P1) showed bilateral lung diffuse lesions with opacification. **c**: Gastroenterological endoscope examination of P2 demonstrated multiple ulcers of the oropharynx (1), small intestine (2), colon (3) and rectum (4). **d**: Morphological examination of bone marrow (P6) revealed abnormal hematopoiesis in granulocyte and erythroid series to different extents. Vacuolar degeneration (1), binucleated (2) or giant (3) granulocytes were observed in granulocyte series. Erythroid series were active proliferation, megaloblastic change and occasionally positive PAS staining (4)

Within a few months after birth, these patients developed conditions like pneumonia, diarrhea, thrush, and otitis media, and one patient presented with omphalitis shortly in the early stage. *Acinetobacter, Enterobacter cloacae, Flavobacterium menigosepticum* and *Haemophilus influenzae* were generally detected from the secretion of lower respiratory tracts via bacterial culture. *Salmonella* was cultured in the stool of two patients with diarrhea. *Parainfluenza virus* was also detected repeatedly from sputum in P1 and P4. Despite oral antiviral and interferon inhalation therapy, the virus was difficult to clear. Pulmonary CT of P1 showed diffuse interstitial changes (Fig. 2), which highly correlated with viral infection. Metagenomic sequencing of the bronchoalveolar lavage fluid sample in P4 detected *Pneumocystis jiroveci* (reads, 189), which partially caused the extensive lung involvement.

#### Autoimmune and other characteristics

Patients may also present with noninfectious manifestations. Routine blood analysis revealed low platelet counts in 3 patients and increased counts after intravenous immune globulin (IVIG) infusion. Other blood system abnormalities, such as leukopenia, occurred in two children. A laboratory examination of P6 suggested pancytopenia at 26 months, and she was suspected of having “myelodysplastic syndromes” based on a bone marrow aspiration smear. However, a bone marrow biopsy was not performed further. Three cases had skin lesions, and one of these patients (P4) presented with erythroderma and one patient with hypopigmentation. P5 primarily suffered from recurrent eczema and skin peeling, accompanied by an increase of eosinophilic cell count to 2.7–8.1 × 10^9^/L.

Notably, P2 was initially admitted to our hospital due to oral ulcers and intermittent fever. Therapy with multiple antibiotics, such as mepin, vancomycin, SMZ and itraconazole, was ineffective, and only respiratory syncytial virus in sputum was detected as positive. Colonoscopy revealed multiple ulcers (Fig. 2), but no etiology was found in biopsy samples using metagenomic sequencing. After steroid administration, the body temperature was lower, and the oral ulcers were slightly better.

### Immune function evaluation

**Fig. 3:**
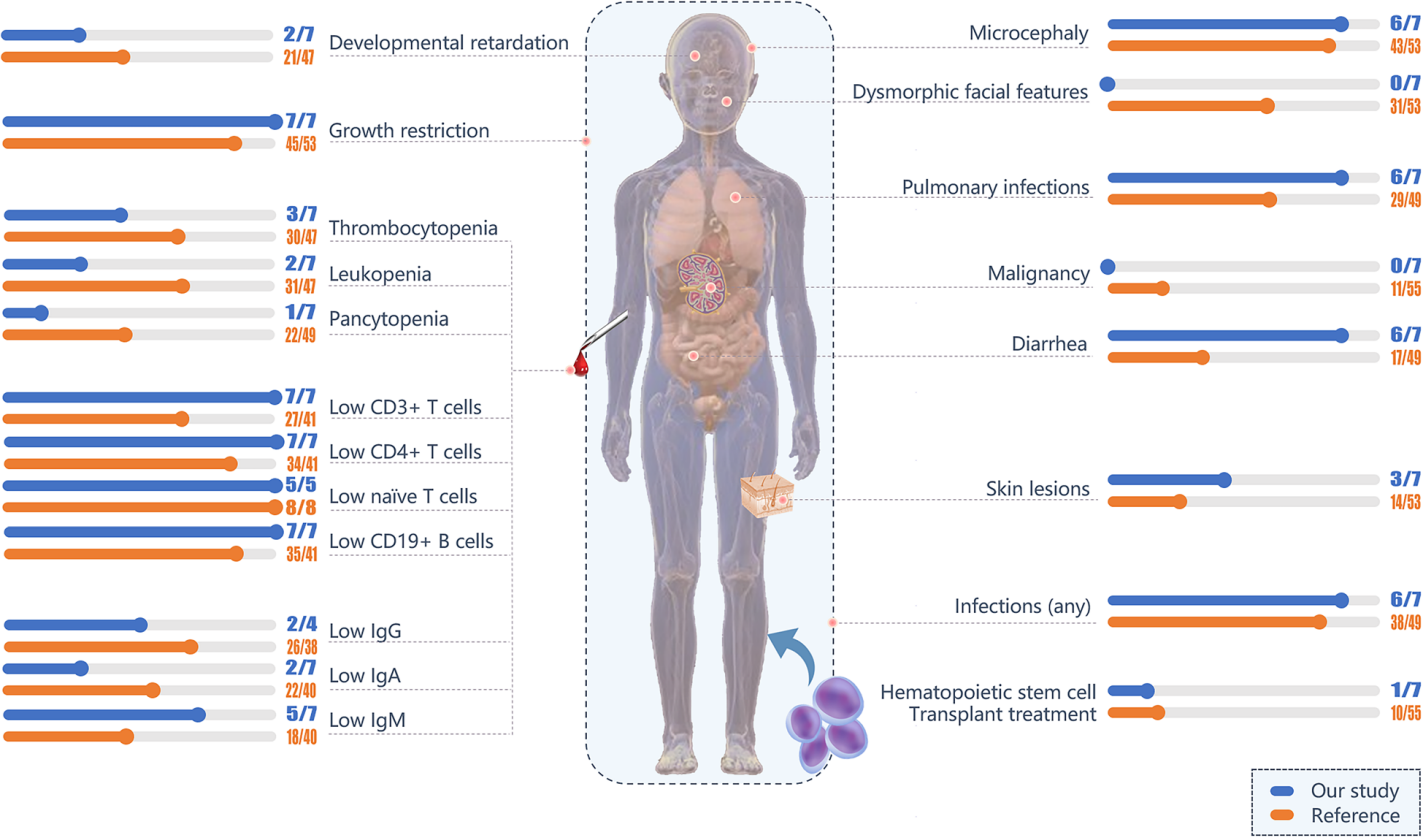
Comparison of clinical presentation and immunophenotyping between this cohort and ~ 55 previously reported patients. Children whose IgG levels were detected after IVIG infusion were not included

Flow cytometric analysis of peripheral blood showed a significant decrease in the absolute numbers of CD19+ B cells and CD3+ T cells (especially CD4+ T cells) but normal or decreased numbers of NK cells (Additional file 1: Table S1). However, the lymphocyte count was decreased slightly in P5, in whom a chimerism test was not performed to exclude maternal-fetal cellular trafficking. Peripheral blood lymphocyte subsets of five patients (P1, P2, P3, P6, and P7) were analyzed in detail. All of these cases exhibited significantly decreased naïve Th cells (CD4 + CD45RA + CD27+) and naïve cytotoxic T cells (CD8 + CD45RA + CD27+) compared to the reference values in healthy children in China [28]. The IgG levels of P5 and P7 were significantly lower than the normal reference value and accompanied by reduced IgM. Other patients had normal levels of IgG, but three patients were tested after IVIG infusion. Comparisons of immune function between this study and previous reports [3–27] are shown in Fig. 3.

### Genetic characteristics

Mutations in the *LIG4* gene were identified in all 7 cases. Patients harbored compound heterozygous mutations that consisted of a missense mutation and a deletion shift mutation. The common missense mutation c.833G > T in exon 2, which causes an amino acid change of Arg to Leu, was identified. In addition, c.1271_1275delAAAGA was found in four patients and became the most frequent deletion mutation. Three other deletion mutations were also detected, specifically c.1144_1145delCT, c.1277_1278delAA and c.1270_1274delAAAAG (Table 2). Mutations c.1144_1145delCT and c.1277_1278delAA were not reported previously and are predicted to be deleterious by Mutation Taster (value 1).

**Table 2:** Mutations of *LIG4 *gene identified in patients

Patients	Zygosity	Exon/Intron	Mutation	Amino acid	Source of variation
P1	Compound heterozygous	Exon2	c.833G>T	p.R278L	Maternal
		Exon2	c.1271_1275delAAAGA	p.K424Rfs*20	Paternal
P2	Compound heterozygous	Exon2	c.833G>T	p.R278L	Paternal
		Exon2	c.1144_1145delCT	p.L382Efs*5	Maternal
P3	Compound heterozygous	Exon2	c.833G>T	p.R278L	Maternal
		Exon2	c.1277_1278delAA	p.E426Gfs*19	Paternal
P4	Compound heterozygous	Exon2	c.833G>T	p.R278L	Paternal
		Exon2	c.1271_1275delAAAGA	p.K424Rfs*20	Maternal
P5	Compound heterozygous	Exon2	c.833G>T	p.R278L	NA
		Exon2	c.1270_1274delAAAAG	p.K424Rfs*20	NA
P6	Compound heterozygous	Exon2	c.833G>T	p.R278L	NA
		Exon2	c.1271_1275delAAAGA	p.K424Rfs*20	NA
P7	Compound heterozygous	Exon2	c.833G>T	p.R278L	Paternal
		Exon2	c.1271_1275delAAAGA	p.K424Rfs*20	Maternal

The most frequent genotype in our cohort was p.R278L/p.K424Rfs*20, which was found in five children. Further comparison with p.R814X/p.K424Rfs*20 reported in previous studies [4, 23] revealed a small difference in phenotype (Additional file 2: Table S2). Facial dysmorphism were more common in patients with genotype p.R814X/p.K424Rfs*20 (7/7) than genotype p.R278/p.K424Rfs*20 (0/9) [3, 19, 24]. Patients with p.R814X/p.K424Rfs*20 had primarily IgG deficiencies, and patients with p.R278/p.K424Rfs*20 were accompanied by a decrease in IgA and IgM levels.

### Treatment and outcome

These patients underwent antibiotic and symptomatic treatment. The choice of empirical antibiotics was primarily based on the immune and infection characteristics and included bacteria, fungi and pneumocystis. Intravenous immunoglobulin was infused as supportive treatments depending on the response. However, most patients (5/7) died or gave up on treatment without transplantation due to severe infection at early stages of the disease. Only P1 received an HLA-matched unrelated HSCT using umbilical cord blood at 29 months. At the time of HSCT, the child had severe pulmonary infection and watery diarrhea with stools 4–6 times per day. Fludarabine and Busulfex were initiated as the conditioning regimen, and cyclosporine A (CsA) was used as GvHD prophylaxis. After transplantation, the child suffered from recurrent fever and aggravated cough. *Flavobacterium menigosepticum* and *Parainfluenza virus 3* were detected in the sputum. Despite supportive treatments with multiple antibiotics and immunoglobulin replacement therapy, this child became worse. Unfortunately, he died half a month later because of respiration-circulation failure and severe inflammation with unsuccessful engraftment (chimerism 0.11% at 7 days).

## Discussion

DNA ligase IV deficiency is a rare primary immunodeficiency that was first reported by Plowman in 1990 [29]. Since then, 55 patients (44 non-Chinese and 11 Chinese) were reported in PubMed Medline with a broad spectrum of clinical features [3–27]. We described the clinical and laboratory features of 7 patients with *LIG4* mutations in a Chinese cohort.

Our study found no obvious family history, except for P2 and P5. Both mothers had abnormal pregnancies with embryo growth arrest. DNA ligase IV is essential for embryonic viability, and knockout mice died at an early embryonic stage [30]. The six patients in this cohort presented with significant microcephaly, and all seven patients had growth restriction, which confirms that these two manifestations are the most prominent features of LIG4 syndrome, as reported previously. Therefore, LIG4 syndrome may be considered when patients present with repeated infection and a head circumference more than 3 SD below the population mean. Developmental delay also occurred in two of our patients. The impairment in expressive language skills was predominant in LIG4 patients [4]. It is necessary to perform early intelligence assessment on patients with microcephaly, even if head MRI shows no apparent abnormality.

Although facial dysmorphism, such as “bird-like” or “Seckel syndrome-like”, are always observed in LIG4 patients, none of our patients had these descriptions in their medical history. Other symptoms, including thrombocytopenia, leukopenia, pancytopenia, and skin lesions, were observed in our patients, except for bone abnormalities. Three patients developed thrombocytopenia. An elevated number of platelets after IVIG infusion suggested potential autoimmune factors, although the results of a routine autoantibody test was negative. One patient manifested as pancytopenia at the age of 2 years, and this patient was suspected of having “myelodysplastic syndromes” (MDS) based on a bone marrow aspiration smear. Most of the patients were at a very young age, so thrombocytopenia may have been more evident than cytopenia. Pancytopenia may mark progression to bone marrow failure, and MDS was reported previously [12]. The pathogeny of MDS was not thoroughly elucidated, but reduced telomere length was found in LIG4 syndrome, which is analogous to other genetically unstable diseases, such as Fanconi anemia and dyskeratosis congenita. We suspected that the accumulation of DSBs in myeloid progenitors led by environmental or infectious triggers and/or autoimmune disorders, may also contribute to bone marrow failure.

Notably, P2 presented with recurrent fever, and colonoscopy indicated extensive intestinal ulceration. To the best of our knowledge, this report is the first patient with an IBD-like phenotype. In previously reported cases, only one patient with LIG4 syndrome was documented to present a phenotype that mimicked Bechet’s disease (BD) [26], and one female patient who was diagnosed with IBD harbored a heterozygous stop-gain (p.R814X) variant in the LIG4 gene [31]. IBD-like immunopathology is a common finding in patients with complex defects in T- and B-cell function, such as Wiskott Aldrich syndrome (WAS) and atypical SCID [32] or Omenn syndrome. Although no further information was found, it seems reasonable to assume the LIG4 gene as a candidate gene for IBD [33]. An abnormal immune reaction in LIG4 patients is likely a contributor. Susceptibility to malignancy is common in disorders influencing double-strand DNA break damage repair. Eleven patients were reported [3, 5–7, 9, 11, 15, 18, 19, 26] whose median onset age of malignancy was 4 years old. However, no malignancy occurred in our study, likely because of the early death of these patients.

NHEJ is also important for immune development. Therefore, LIG4 patients manifest as (severe) combined immunodeficiency. Among the reported cases, approximately three quarters of patients suffered from recurrent infection with varying degrees of severity. In our case series, recurrent pneumonia and diarrhea were the most common symptoms. Pathogens, including *Streptococcus pneumoniae*, *Haemophilus influenzae*, *CMV*, *Candida albicans*, *Salmonella typhimurium*, and *EBV*, are frequently detected in LIG4 patients. Exhibiting a slight difference, the *Acinetobacter, Parainfluenza virus* and *Salmonella* infections are also prominent. The immunological detection showed a profound T (especially CD4+ T) and B lymphocytopenia with hypogammaglobulinemia in most of our patients. One patient just showed slightly decreased lymphocytes, and we suspect that this may reflect compensatory mechanisms of the proliferation of T cells or chimerism, such as maternal-fetal cellular trafficking. Further analysis of lymphocyte subsets in our patients revealed that the proportion of naïve CD4+ and naïve CD8+ T cells were markedly decreased, and memory T cells were increased, which is consistent with the three siblings reported by Felgentreff et al. [21]. The decreased naïve T cells may lead to ineffective resistance to primary infection. All patients in this cohort had significantly reduced B cell counts, but some patients had normal immunoglobulin levels, likely due to the ability of the remaining B cells to develop into plasma cells and produce antibodies.

Nearly 85% of reported patients carry compound heterozygous mutations in the LIG4 gene, and the most frequent alleles are R814X and K424fs in non-Chinese cases [3]. Mutation K424fs causes a premature stop codon 20 aa downstream, and the protein expression level in skin fibroblasts is notably reduced [8]. Truncating mutation R814X, which is at the XRCC4 binding domain, retained 10% ~ 15% residual double-strand ligation activity [6]. However, the slight difference is that two mutations R278L and K424fs account for most Chinese patients [19, 25]. The R278L mutation resides in the vicinity of the ATP-binding site. We predict that the function mechanism may be similar to the previously reported R278H mutation at the same position. Interaction between the ligase and XRCC4 is not affected, but the enzyme–adenylate complex formation is severely impaired, which results in the ~ 10% residual ligase activity [34, 35]. In all LIG4-mutated patients, the most prevalent genotypes are p.R814X associated with another truncating mutation, especially p.R814X/p.K424Rfs*20, and p.K424Rfs*20 associated with mutations near active site (K273) is another frequent genotype [23]. The phenotypes of these two genotypes are also slightly different from each other. Notably, p.K424Rfs*20/p.R278L is the only genotype seen in the Chinese population [19], and two patients with p.K424Rfs*20/p.R278H were reported in other countries [3, 24], which highlights the significance of the genotype p.K424Rfs*20/p.R278. Further comparison with the genotype p.R814X/p.K424Rfs*20 [4, 23], suggests that p.K424Rfs*20/p.R278 leads to milder dysmorphism, but more significant IgA/IgM deficiency. Hypomorphic mutations in ligase IV, such as R278H, may allow normal development but confer marked radiosensitivity, as reported in previous studies [35]. Decreased IgA/IgM levels may be associated with early clinical digestive symptoms, similar to the disease selective IgA deficiency. Patients with p.K424Rfs*20/p.R278 are at higher risk of severe infections. These findings deepen our understanding of genotype-to-phenotype correlations.

The treatment of LIG4 syndrome primarily includes antibiotic prophylaxis, immunoglobulin substitution, transfusion support and avoiding unnecessary exposure to ionizing radiation [1]. Hematopoietic stem cell transplantation (HSCT) has been performed in 10 patients [8, 9, 12–14, 17, 22, 23, 25], six of whom were successful, but the others died because of different complications, primarily infections. Notably, microcephaly or neurodevelopmental delay in these patients cannot be cured with HSCT. Hematopoietic stem cell transplantation requires cytotoxic agents, especially CsA [36], which may induce DSBs and be harmful for patients with this syndrome. Therefore, personalized transplant conditioning should be performed carefully in these patients. Only one patient in our study underwent HSCT and died due to severe infection. Other patients received supportive treatment. Notably, P2 was treated with glucocorticoids due to persistent fever and IBD. Immunosuppressant was also used in the reported patient who presented with a BD-like phenotype.

## Conclusions

In summary, we reported the clinical manifestations and treatment of seven Chinese patients with LIG4 syndrome. The finding of the IBD-like phenotype may expand the phenotypic spectrum of this disease. R278L and K424fs were two common mutations, and p.K424Rfs*20/p.R278L was the only genotype seen in the Chinese population. Genotype p.K424Rfs*20/p.R278 might lead to milder dysmorphism but more significant IgA/IgM deficiency compared to p.R814X/p.K424Rfs*20. Genotype-to-phenotype correlations were further understood.

## Supplementary information

**Additional file 1: Table S1.** Immune index of patients with LIG4 mutations.

**Additional file 2: Table S2.** Clinical and immunophenotype comparison between genotypes p.R814X/p.K424Rfs*20 and p.R278/p.K424Rfs*20.

## Data Availability

The datasets used and/or analysed during the current study are all included within the article and available from the corresponding author on reasonable request.

## Abbreviations

BD: Behçet’s disease
DSBs: DNA double strand breaks
GvHD: Graft-versus-host disease
HLA: Human leukocyte antigen
HSCT: Haematopoietic stem cell transplant
IBD: Inflammatory bowel disease
IVIG: Intravenous immune globulin
LIG4: DNA Ligase IV
MDS: Myelodysplastic syndromes
NGS: Next-generation sequencing
NHEJ: Nonhomologous end-joining
UCBSCT: Umbilical cord blood stem cell transplantation
